# Identification of the Key Genes Involved in Proline-Mediated Modification of Cell Wall Components in Rice Seedlings under Trivalent Chromium Exposure

**DOI:** 10.3390/toxics12010004

**Published:** 2023-12-19

**Authors:** Abid Ullah, Yu-Juan Lin, Hua Zhang, Xiao-Zhang Yu

**Affiliations:** College of Environmental Science & Engineering, Guilin University of Technology, Guilin 541004, China; 2023009@glut.edu.cn (A.U.); yujuan@glut.edu.cn (Y.-J.L.); zhanghua@glut.edu.cn (H.Z.)

**Keywords:** cell wall, rice, Cr stress, proline, pectin pathway

## Abstract

Chromium (Cr) toxicity exerts a detrimental effect on various physiological, biochemical, and molecular attributes of plants including the structure and functions of cell walls. On the other hand, the exogenous application of proline (Pro) is a beneficial strategy to overcome Cr toxicity. Therefore, it is a novel strategy to find the key genes associated with cell wall composition in rice under trivalent Cr with/without Pro application. A total of 203 genes were activated in the four cell wall biosynthesis pathways under chromium stress, namely cellulose (60), hemicellulose (57), lignin (35), and pectin (51). Based on the expression abundance of microarrays, the number of differentially expressed genes, and the expression level of genes, the lignin pathway was a crucial pathway in response to Cr treatments, followed by the cellulose pathway. Through the estimation of gene expression variation factors between ‘Cr’ and ‘Cr+Pro’ treatments, *OsUGP1*, *OsBGLU24*, *OsBGLU29*, *OsBGLU33*, *OsBMY1*, and *OsBMY2* in the cellulose pathway; *OsXTH9*, *OsXTH10*, *OsXTH16*, *OsGAUT3*, *OsGAUT19*, *OsGAUT28*, *OsXTH1*, *OsGAUT12*, and *OsGAUT21* in the hemicellulose pathway; *OsPAL3*, *OsPAL3*, *OsPOX1*, and *OsPRX77* in the lignin pathway; and *OsPME25*, *OsPGL27*, *OsPME26*, *OsPGL9*, and *OsPLL12* in the pectin pathway are the key genes involved in cell wall modification during Cr exposure with exogenous Pro application. The Pro-mediated activation of these genes could be crucial players in modifying the cell wall structure and composition of rice plants under Cr stress, which needs to be further clarified.

## 1. Introduction

The dawn of industrialization rescued human beings from food scarcity, but its effluents produced several primary and secondary pollutants that are currently being faced by all living organisms. Among these, heavy metals (HM) are well-known environmental pollutants owing to their toxicity. Chromium is one of the toxic heavy metals which pose hazardous effects on plants and animals [1]. It is chiefly produced by several anthropogenic activities such as mineral degradation, metal refining, effluents of textile dyes, paper and paint industries, coal burning, urban sewage, and agriculture chemicals [2,3]. The accumulation of Cr in environmental matrixes, i.e., soil and water, increases their chances of interaction with plants and subsequently their entrance into the food chain [4]. The entrance of Cr into the human body via the food chain poses serious health problems including lung and skin cancer and is therefore included in the top 20 hazardous materials [2]. A higher concentration of Cr significantly affects various morphophysiological and biochemical parameters in plants, i.e., reduces seed germination, photosynthesis, respiration, and plant biomass, and may lead to their death [5].

During the long-term intercourse of plants with the environment, plants have developed several sophisticated mechanisms to cope with HM stress including (1) the use of cell walls to intercept metal ions outside the cell and reduce their uptake and translocation; (2) the use of efflux transporters to remove HM ions from the cell; (3) the chelation and sequestration of metal ions into subcellular compartments; and (4) the activation of antioxidant systems [6]. This shows that the cell wall is the front-line defense mechanism of plants against HM stress. The thick cell walls act as a barrier that limits HMs from entering into the protoplasm as well as serving as a compartment for storing the metal [7]. In the case of Cr, the uptake and translocation are variable in different plant species; however, a higher concentration of Cr was observed in roots than aerial parts [8,9], suggesting the crucial role of the cell wall in Cr uptake and translocation. In fact, hexavalent and trivalent are the most stable species in the Cr family [10], and both species are bioavailable for plants, but with differences in uptake and distribution in plants [9].

In addition to the cell wall, plants also accumulate several compatible molecules (amino acids) to protect plants from the deleterious effects of HMs through the detoxification of reactive oxygen species (ROS), osmotic adjustment, metal chelation, protein stabilization, and membrane integrity protection [11]. In addition, it is noted that the exogenous application of these molecules further strengthens the defense mechanism of plants against HM stresses [12]. Proline, an amino acid, acts as a metabolic signaling molecule that regulates development and the stress response in plants. In addition, Pro induces stress-protective proteins and the expression of stress-associated genes [13]. In one case, the exogenous application of Pro reduced the adverse effects of Cr in rice plants by regulating glyoxalase I- and II-related genes [14]. Moreover, Pro takes part in the biosynthesis of hydroxyproline-rich glycoproteins (proline-rich proteins, PRPs) that are present in the cell wall matrix [15]. It has been found through a study that the overexpression of *OsPRP3* in rice enhanced cell wall integrity and consequently improved cold tolerance [16]. In this regard, the expression profile of genes associated with cell wall composition in rice plants under trivalent chromium (Cr(III)) stress with or without exogenous Pro was investigated with the following objectives: (1) to calculate the expression profile of genes activated in four cell wall pathways; (2) to estimate the gene expression variation factors (*GEVFs*) under ‘Cr’ treatments due to the application of exogenous Pro; and (3) to identify the key genes involved in the Pro-mediated modification of cell wall composition of rice seedlings under Cr exposure.

## 2. Materials and Methods

### 2.1. Plant Materials, Growth Conditions, and Treatments

Rice seeds (*Oryza Sativa* L. cv. XZX45) were granted by the Hunan Academy of Agricultural Sciences, Hunan, China. The seeds were cultivated in small pots containing river sand. Afterward, the pots were kept in an artificial climate chamber to germinate seeds under controlled conditions (25 ± 1 °C, 24 h light with 20,000 lux, and 60 ± 2% humidity). After 16 days of growth with irrigation of a modified-8692 nutrient solution, seedlings of similar sizes were rinsed with an ion buffer solution to clean the root surface from any additional ions. To acclimatize rice seedlings for treatments in hydroponics, seedlings were pretreated with the modified nutrient solution for 12 h. The pretreated rice seedlings were then subjected to Cr stress for 2 days in the following manner.

Cr treatment: 16-day-old rice seedlings were placed in a nutrient solution containing different concentrations of Cr(NO_3_)3.9H_2_O, i.e., 12.0, 24.0, and 40.0 mg/L, with the 0 control group. This group is represented by ‘Cr’.

Cr treatment with proline: 16-day-old rice seedlings were first exposed to a nutrient solution containing 1 mM proline for 12 h and then the Pro-treated seedlings were subjected to Cr(NO_3_)3.9H_2_O of different concentrations, i.e., 12.0, 24.0, and 40.0 mg/L, with the Pro-treated control group. This group is represented by ‘CrP’.

The Cr concentrations were based on a previous study [17]. All chemical reagents, including Cr(NO_3_)3.9H_2_O used in this study, were of analytical grade. All flasks were wrapped in aluminum foil to inhibit algal growth and reduce evaporation to a minimum level.

### 2.2. Microarray Analysis

Rice seedlings were collected after 2 days of ‘Cr’ and ‘CrP’ treatments to wash with deionized water and separate their shoots and roots. Subsequently, total RNA was isolated from both the roots and shoots of treated rice seedlings as described in Yu et al. [17]. Briefly, Ultrapure RNA Kit [(DNase I) (CWBio, Taizhou, China)] was used to extract total RNA from the 0.2 g of rice tissues. To prevent any genomic DNA contamination, the extracted RNA was initially treated with DNase I and then purified using RNeasy MinElute Cleanup kit (Qiagen, Helden, Germany), respectively.

The Agilent 4X44K rice microarray with oligonucleotide 44,000 probes was used in this study. Microarray hybridization, washing, staining, scanning, and data processing were carried out by Shanghai Biotechnology Corporation (Shanghai, China). Hybridization signal data were deposited into GeneSpring Software version 12.5 (Agilent Technology, Santa Clara, CA, USA) for screening of differentially expressed genes (DEGs). The DEGs data were normalized by Quantile algorithm. The maximum level for the selection of DEGs was fixed as a *p* value < 0.05 and the fold change ratio between the non-treated and treated samples was <0.5 or >2.0 [17].

### 2.3. RT-qPCR Analysis

To verify the changes in gene expression identified through microarray analysis, RT-PCR was conducted using the same RNA samples. In this PCR verification, 28 genes were randomly selected. The sequence of forward and reverse primers with their gene symbols, MSU ID, amplicon size, and associated enzymes are listed in Table S1. The RT-qPCR cycling conditions were set up as follows for 40 cycles: denaturation at 95 °C for 10 s, annealing at 58 °C for 30 s, and extension at 72 °C for 32 s. The RT-qPCR was performed using Applied Biosystems 7500 RT-qPCR system and SYBR green chemistry. *OsGAPDH* (LOC_Os08g03290.1) was used as a housekeeping standard gene. The relative expression of each gene was calculated using the standard 2^−ΔΔCT^ method [18].

### 2.4. Calculation of Gene Expression Variation Factors (GEVFs)

The gene expression variation factors (*GEVFs*, %) were determined by comparing the fold change of gene expression of the selected genes in ‘Cr’ treatments versus ‘CrP’ treatments.

$$GEVFs=\frac{FC_{\left(CrP\right)}-FC_{\left(Cr\right)}}{FC_{\left(Cr\right)}}\times 100\%)$$

where *FC*_(*CrP*)_ represents the fold changes in the expression of the genes from the ‘CrP’ treatments, while *FC*_(*Cr*)_ represents fold changes in the expression of the genes from the ‘Cr’ treatments.

### 2.5. Data Analysis

Four independent biological replicates were used. To evaluate the statistical significance of the variance between the control and treatment groups, Tukey’s multiple comparison tests were performed at a significance level of *p* < 0.05. The *GEVF* threshold was set at >25% or <−25% to indicate whether a gene was being promoted or repressed, respectively, which means the difference in expression levels was significant (*p* < 0.05).

## 3. Results

### 3.1. Identification of Genes Involved in Cell Wall Components

**Figure 1:**
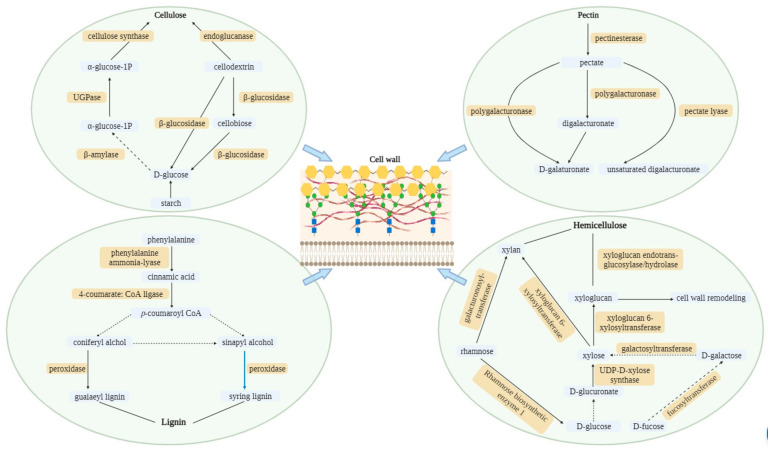
Activated pathways of cell wall components (cellulose, hemicellulose, lignin, and pectin) in rice under Cr stress. The dotted lines represent the omission of some steps.

Initially, the KEGG pathway database (https://www.kegg.jp/kegg/pathway.html; accessed on 12 April 2023) was used to find the cell wall component pathways in rice plants, namely cellulose, hemicellulose, lignin, and pectin. Next, the activated genes encoding these components were searched in the following databases: RGAP (http://rice. plantbiology.msu.edu/analyses_search_blast.shtml; accessed on 20 April 2023), NCBI (https://www.ncbi.nlm.nih.gov/; accessed on 20 April 2023), and RAPDB (http://rapdb.dna.affrc.go.jp/; accessed on 20 April 2023). Cellulose, hemicellulose, lignin, and pectin pathways were activated under Cr stress with or without Pro application (Figure 1). Based on the data of microarray analysis, a total of 203 genes associated with the four pathways of plant cell wall components, i.e., cellulose (60), hemicellulose (57), lignin (35), and pectin (51), were detected. All the data related to these genes, i.e., enzyme name, gene name, chromosome locus, and Cr-concentration-dependent up/downregulation in roots and shoots, are provided in Supplementary Data S1.

### 3.2. Differentially Expressed Genes Associated with Cell Wall Pathways

**Figure 2:**
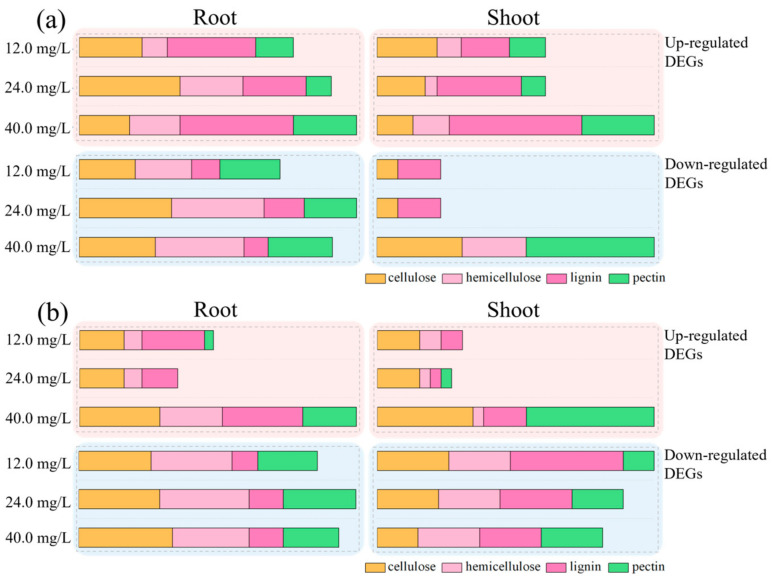
The DEGs in rice seedlings under different concentrations of (**a**) Cr and (**b**) CrP.

The total number of DEGs detected in rice seedlings under the three effective concentrations of Cr with and without Pro treatment are listed in Table S2. The number of upregulated DEGs (associated with cellulose, hemicellulose, lignin, and pectin pathways) detected in rice roots under 12.0, 24.0, and 40.0 mg/L of ‘Cr’ treatments were 17 (5, 2, 7, 3), 20 (8, 5, 5, 2), and 22 (4, 4, 9, 5), respectively. At the same time, downregulated DEGs associated with cellulose, hemicellulose, lignin, and pectin pathways in rice roots treated under 12.0, 24.0, and 40.0 mg/L of ‘Cr’ were 50 (14, 14, 7, 15), 69 (23, 23, 10, 13), and 63 (19, 22, 6, 16), respectively. In shoots, the upregulated DEGs that are categorized in cellulose, hemicellulose, lignin, and pectin pathways under 12.0, 24.0, and 40.0 mg/L ‘Cr’ treatments were 14 (5, 2, 4, 3), 14 (4, 1, 7, 2), and 23 (3, 3, 11, 6), respectively. On the other hand, downregulated DEG numbers that are categorized in cellulose, hemicellulose, lignin, and pectin pathways in rice shoots treated with 12.0, 24.0, and 40.0 mg/L ‘Cr’ treatments were 3 (1, 0, 2, 0), 3 (1, 0, 2, 0), and 13 (4, 3, 0, 6), respectively (Figure 2; Supplementary Data S1).

Under ‘CrP’ treatments, the number of upregulated DEGs that are associated with cellulose, hemicellulose, lignin, and pectin pathways in rice roots under 12.0, 24.0, and 40.0 mg/L ‘Cr’ treatments were 15 (5, 2, 7, 1), 11 (5, 2, 4, 0), and 31 (9, 7, 9, 6), respectively, while the numbers of downregulated DEGs associated with cellulose, hemicellulose, lignin, and pectin pathways in rice roots under 12.0, 24.0, and 40.0 mg/L ‘CrP’ treatments were 56 (17, 19, 6, 14), 65 (19, 21, 8, 17), and 61 (22, 18, 8, 13), respectively. The numbers of upregulated DEGs in rice shoots that are categorized in cellulose, hemicellulose, lignin, and pectin pathways under 12.0, 24.0, and 40.0 mg/L ‘CrP’ treatments were 8 (4, 2, 2, 0), 7 (4, 1, 1, 1), and 26 (9, 1, 4, 12), respectively. On the other hand, downregulated DEG numbers that are categorized in cellulose, hemicellulose, lignin, and pectin pathways in rice shoots treated with 12.0, 24.0, and 40.0 mg/L ‘CrP’ treatments were 27 (7, 6, 11, 3), 24 (6, 6, 7, 5), and 22 (4, 6, 6, 6), respectively (Figure 2; Supplementary Data S1).

These data reveal that more genes were up/downregulated in roots compared with shoots under ‘Cr’ and ‘CrP’ treatments. In addition, the number of upregulated genes was generally increased with an increase in Cr dose in both roots and shoots of rice seedlings. In shoots, the number of upregulated genes was 41 and 51, while the number of downregulated genes was 19 and 73 in ‘Cr’ and ‘CrP’ treatments, respectively. In roots, there was no significant difference recorded at 12.0 and 24.0 mg Cr/L; however, Pro application with 40.0 mg/L Cr treatments increased the number of upregulated genes compared with ‘Cr’ treatments alone. Among the upregulated genes, the highest number of genes was associated with lignin in both roots and shoots, while the lowest number of downregulated genes was associated with the same component, i.e., lignin in the roots (Figure 2; Supplementary Data S1).

### 3.3. PCR Verification to Validate the Reliability of Rice Microarray

**Figure 3:**
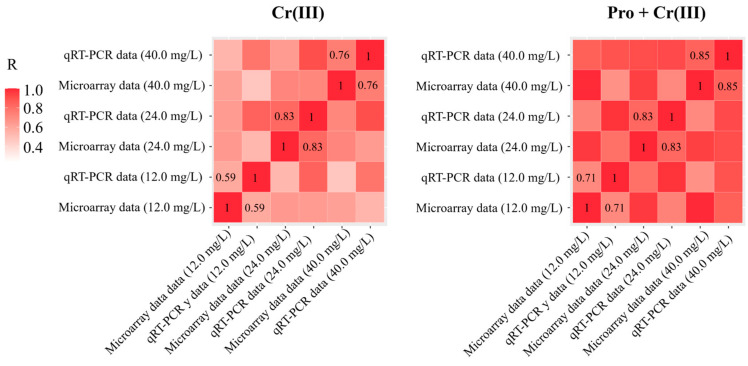
Comparison of fold changes measured using the Agilent 4 × 44 K rice microarray with data from the qRT-PCR for 28 selected genes associated with the four pathways of cell wall biosynthesis in shoots of rice seedlings under ‘Cr’ and ‘CrP’ treatments.

To validate the data of microarray analysis, log-fold changes were calculated to determine the log ratio of genes associated with the four pathways responsible for cell wall biosynthesis under ‘Cr’ and ‘CrP’ treatments. All 28 genes showed almost similar expression patterns in qRT-PCR analysis as shown in the microarray analysis, which demonstrates the reliability of the microarray data. The reliability of microarray data was judged by the R-value, which indicates the significant correlation between the expression analysis of PCR and microarray data. Although changes in qRT-PCR data were slightly different from the results of microarray analysis (Figure 3), this discrepancy could be attributed to technical differences in analysis and normalization methods.

### 3.4. Specific Genes Activated in Cell Wall Biosynthesis

**Figure 4:**
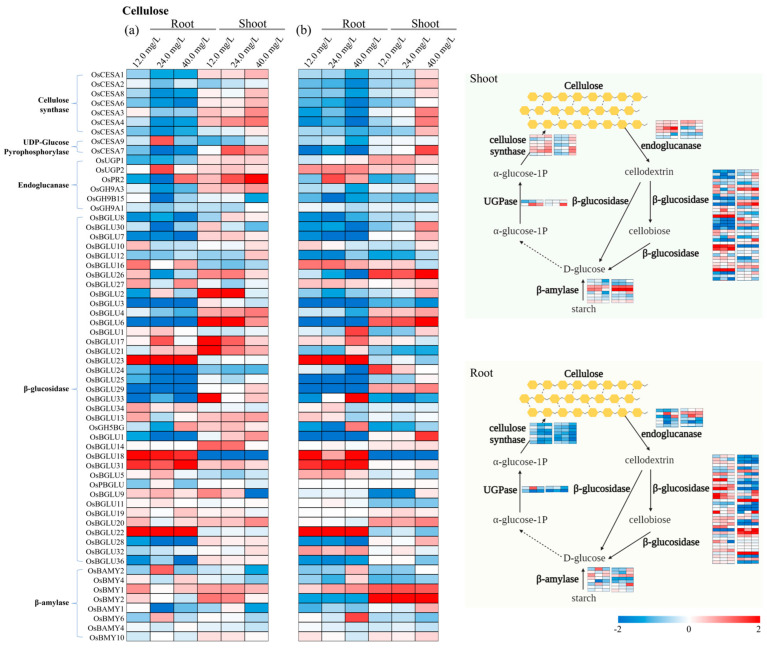
Expression pattern of the identified genes associated with cellulose pathway of cell walls in roots and shoots of rice seedlings under (**a**) ‘Cr’ and (**b**) ‘CrP’ treatments. Genes are listed on *y*-axis and experimental conditions on *x*-axis. Red and blue shadings represent higher and lower relative expression levels, respectively.

**Figure 5:**
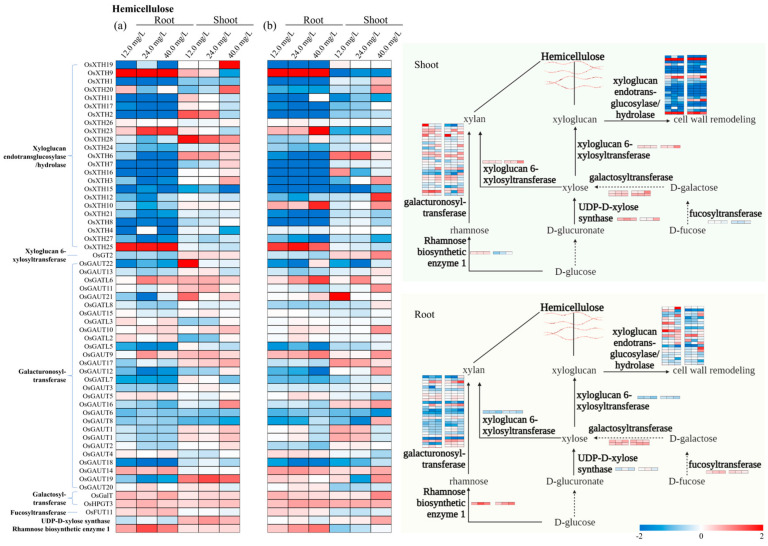
Expression pattern of the identified genes associated with hemicellulose pathway of cell walls in roots and shoots of rice seedlings under (**a**) ‘Cr’ and (**b**) ‘CrP’ treatments. Genes are listed on *y*-axis and experimental conditions on *x*-axis. Red and blue shadings represent higher and lower relative expression levels, respectively.

**Figure 6:**
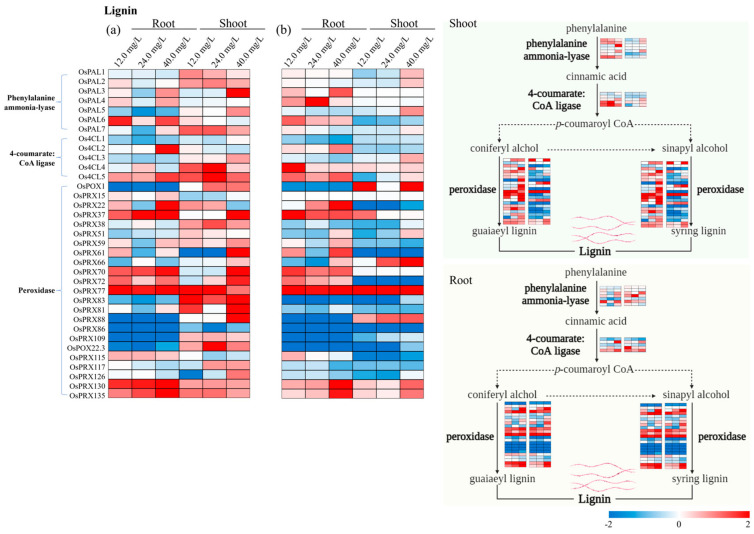
Expression pattern of the identified genes associated with the lignin pathway of cell walls in roots and shoots of rice seedlings under (**a**) ‘Cr’ and (**b**) ‘CrP’ treatments. Genes are listed on *y*-axis and experimental conditions on *x*-axis. Red and blue shadings represent higher and lower relative expression levels, respectively.

**Figure 7:**
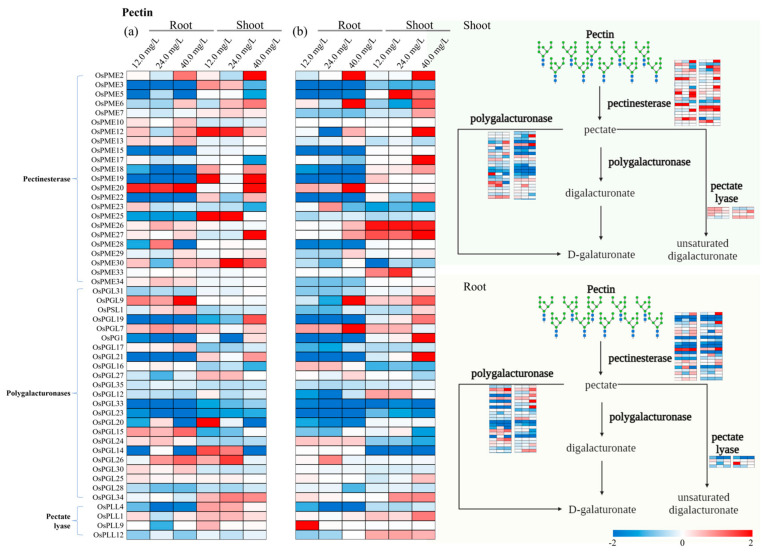
Expression pattern of the identified genes associated with the pectin pathway of cell walls in roots and shoots of rice seedlings under (**a**) ‘Cr’ and (**b**) ‘CrP’ treatments. Genes are listed on *y*-axis and experimental conditions on *x*-axis. Red and blue shadings represent higher and lower relative expression levels, respectively.

The expression pattern of cell wall biosynthesis genes associated with cellulose, hemicellulose, lignin, and pectin pathways in rice tissues under various treatments of ‘Cr’ and ‘CrP’ are presented through heat maps (Figure 4, Figure 5, Figure 6 and Figure 7). Among the genes associated with the cellulose pathway, *OsBGLU22*, *OsBGLU23*, and *OsBGLU31* were the upregulated genes in roots from both ‘Cr’ and ‘CrP’ treatments (Figure 4). Similarly, *OsBGLU6*, *OsBGLU26*, *OsBMY1*, and *OsBMY2* were the upregulated genes in shoots under ‘CrP’ treatments. *OsXTH29* and *OsXTH25* were the upregulated genes in roots associated with the hemicellulose pathway of cell walls in both ‘Cr’ and ‘CrP’ treatments (Figure 5). In the lignin pathway, *OsPRX37*, *OsPRX70*, and *OsPRX77* in roots, and *OsPRX77* in shoots were the upregulated genes in both ‘Cr’ and ‘CrP’ treatments (Figure 6), while *OsPRX130* and *OsPRX137* were the only upregulated genes observed under ‘Cr’ treatments. Several genes in roots (i.e., *OsPME25* and *OsPGL27*) and shoots (*OsPME26*, *OsPGL9*, and *OsPLL12*) were significantly upregulated under all the treatments of ‘CrP’ compared with the ‘Cr’ exposure alone. There was only one gene (*OsPME20*) that was upregulated under ‘Cr’ treatments; however, there were no commonly upregulated genes in both ‘Cr’ and ‘CrP’ treatments associated with the pectin pathway (Figure 7).

These results suggest that several genes associated with cellulose, hemicellulose, lignin, and pectin pathways of cell walls in both shoots and roots of rice seedlings were upregulated under Cr treatments.

### 3.5. Estimation of Gene Expression Variation Factors (GEVFs)

**Figure 8:**
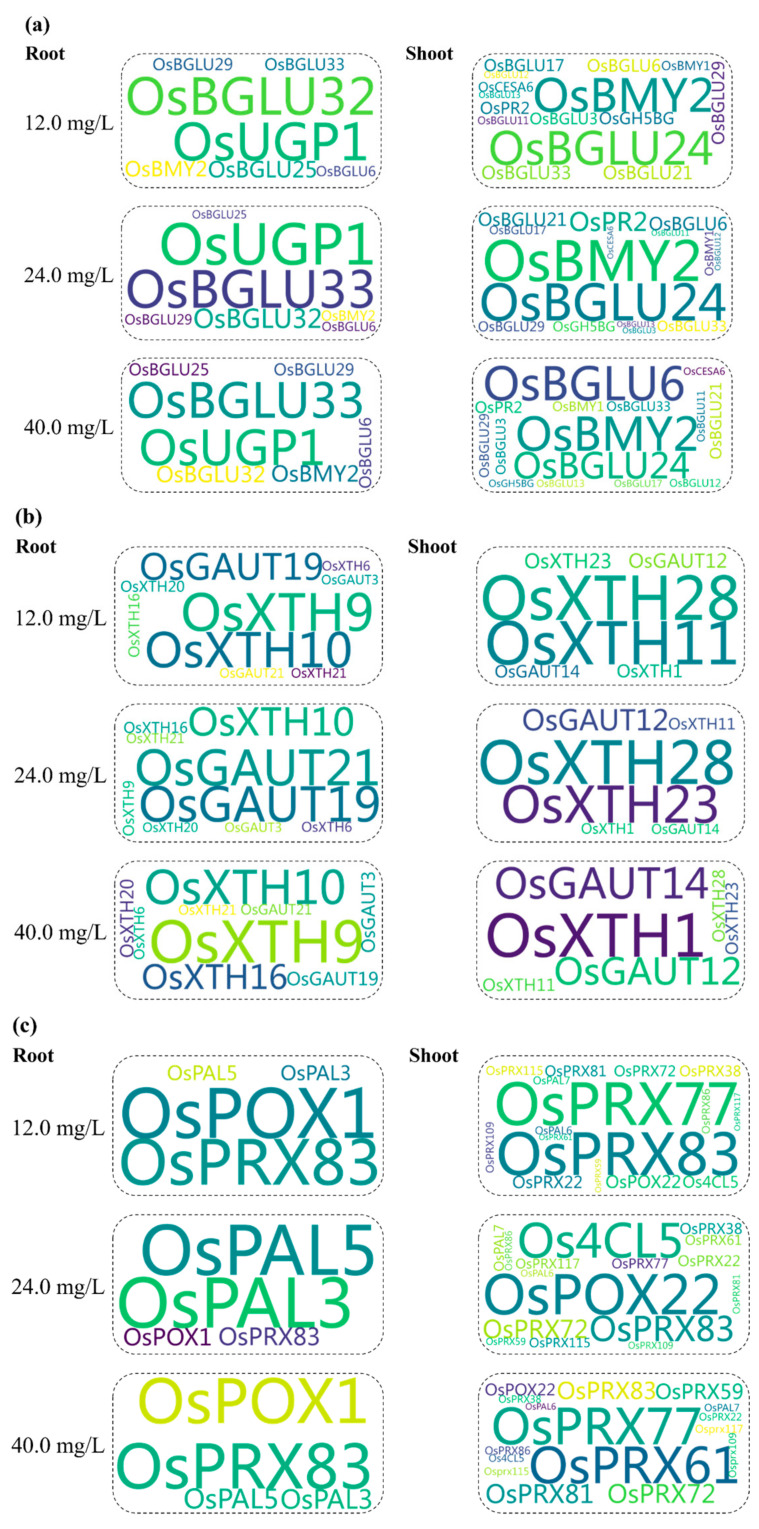

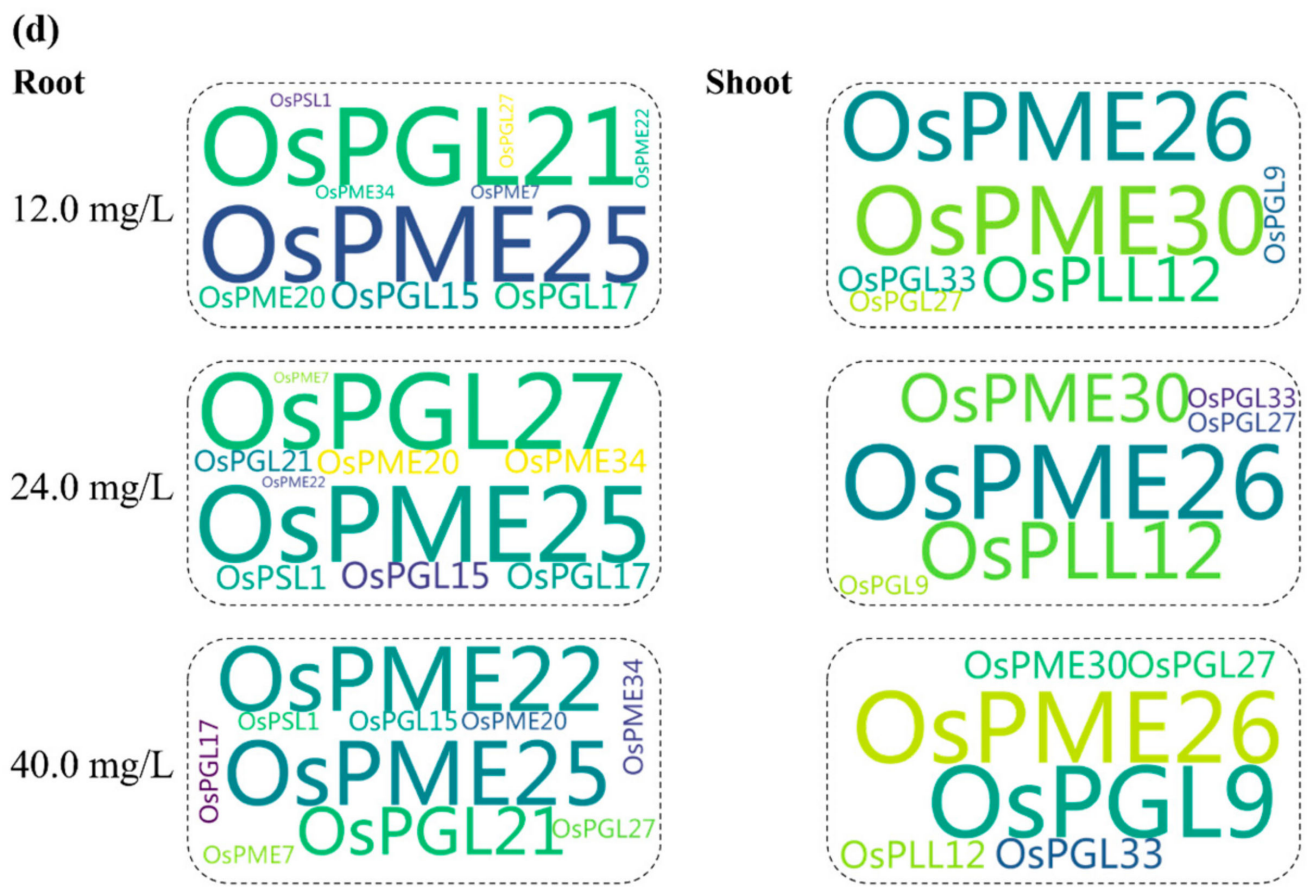
Gene expression variation factors within ‘Cr’ and ‘CrP’ treatments associated with (**a**) cellulose, (**b**) hemicellulose, (**c**) lignin, and (**d**) pectin pathways of cell walls.

The influence of exogenous Pro on expression levels of cell wall biosynthesis genes in rice plants during Cr exposure was calculated by the *GEVFs* (Figure 8). Their values indicate that a few genes associated with cellulose, hemicellulose, lignin, and pectin pathways of cell walls showed significantly higher responses to Pro application. In the cellulose pathway, *OsUGP1* and *OsBGLU32* at 12 mg Cr/L, *OsBGLU33* and *OsUGP1* at 24 mg Cr/L, and *OsBGLU33* and *OsUGP1* at 40 mg Cr/L showed higher *GEVF* values for ‘CrP’ treatments in root tissues of rice seedlings. Additionally, *OsBMY2* and *OsBGLU24* at 12 and 24 mg Cr/L, and *OsBMY2* and *OsBGLU6* at 40 mg Cr/L showed a higher *GEVF* value for ‘CrP’ treatments in shoot tissues of rice seedlings.

In the hemicellulose pathway, *OsXTH9* and *OsXTH10* at 12 and 40 mg Cr/L, and *OsGAUT19* and *OsGAUT20* at 24 mg Cr/L showed a higher *GEVF* value for ‘CrP’ treatments in root tissues of rice seedlings. In shoots, *OsXTH11* and *OsXTH28* at 12 mg Cr/L, *OsXTH23* and *OsXTH28* at 24 mg Cr/L, and *OsXTH1* and *OsGAUT14* at 40 mg Cr/L showed higher *GEVF* values for ‘CrP’ treatments in the hemicellulose pathway of cell walls.

In the lignin pathway of cell walls, *OsPOX1* and *OsPRX83* at 12 mg Cr/L, *OsPAL3* and *OsPAL5* at 24 mg Cr/L, and *OsPOX1* and *OsPRX83* at 40 mg Cr/L showed higher GEVF values for ‘CrP’ treatments in root tissues of rice seedlings. In shoots, *OsPRX77* and *OsPRX83* at 12 mg Cr/L, *OsPOX22* and *OsPCL5* at 24 mg Cr/L, and *OsPRX77* and *OsPRX61* at 40 mg Cr/L showed higher *GEVF* values for ‘CrP’ treatments in the same pathway.

In the pectin pathway of cell walls, *OsPME25* had higher *GEVF* values for ‘CrP’ treatments in roots at all the ECs of Cr, while *OsPGL21*, *OsPGL27*, and *OsPME22* had higher *GEVF* values at 12, 24, and 40 mg Cr/L, respectively. In the same pathway, *OsPME26* had higher *GEVF* values for ‘CrP’ treatments in shoots of rice seedlings at all the ECs of Cr, while *OsPPME30*, *OsPLL12*, and *OsPGL9* had higher *GEVF* values at 12, 24, and 40 mg Cr/L, respectively.

## 4. Discussion

The cell wall is the first cellular organelle of a cell that faces toxic metals (Cr) and acts as a defense wall to protect the inside metabolic organelles [6,7]. In this regard, the identification of key genes associated with cell wall biosynthesis is the first and important step for further studies to improve stress tolerance in all plants. In this regard, microarray analysis was conducted to identify cell wall-associated genes that are activated in rice plants under Cr stress with or without exogenous Pro application. The data of microarray analysis revealed that four pathways of plant cell wall components, i.e., cellulose, hemicellulose, lignin, and pectin, were activated under Cr stress. Several genes associated with these pathways were up/downregulated in the roots and shoots of rice seedlings in response to Cr stress. According to the deferential gene expression data, a higher number of genes were up/downregulated in roots compared with shoots under ‘Cr’ and ‘CrP’ treatments. This reveals that the stress response in roots was higher compared with the shoots. This is most likely due to the higher Cr accumulation in the roots of rice plants. In our previous study, the accumulation of Cr was higher in the roots than in the shoots of rice seedlings [19]. On the other hand, shoots have fever genes than roots that were induced by Cr treatments, because Cr translocation from the roots to the shoots is very limited [20]. The number of upregulated genes increased with the concentration of Cr in both roots and shoots, suggesting that a higher concentration of Cr has a greater effect on rice seedlings, and their response level also increased. Pro application decreased the number of upregulated genes and increased downregulated genes in shoots. In roots, Pro application only increased the number of upregulated genes at 40.0 mg/L Cr treatments. This reveals that Pro application rescued rice shoots from the effects of Cr stress, which consequently downregulated a higher number of genes responsible for several physiological and biochemical processes. However, roots can tolerate a higher concentration of Cr in rice plants and thereby only the highest effective concentration increased the number of upregulated genes for an effective response to Cr stress [21]. It is also noted that the highest number of upregulated genes were associated with lignin (43) in both roots and shoots compared with cellulose (29), hemicellulose (17), and pectin (21). Similarly, the lowest number of downregulated genes was also associated with lignin (23) in the roots compared with cellulose (56), hemicellulose (59), and pectin (44). This suggests the significant role of lignin in cell walls under Cr stress in rice, because lignin polymers include several hydroxyl, carboxyl, methoxyl, and other functional groups that can bind multiple HMs and reduce their entrance into the cytosol [22].

Cellulose is an essential component of the cell wall synthesized by cellulose synthase complexes that are assembled in the Golgi apparatus and then transported to the plasma membrane. Here, they actively synthesize cellulose and organize it into microfibrils that serve as the main load-bearing elements of cell walls [23]. The β-glucosidases (BGLU) enzyme is involved in β-glucan (cellulose) synthesis during cell wall development, pigment metabolism, fruit ripening, and defense mechanisms [24], whereas *OsBGLU32*, *OsBGLU33*, *OsBGLU24*, *OsBGLU25*, and *OsBGLU26* were higher-expressed genes under ‘CrP’ application compared with ‘Cr’ treatments. In addition, *OsBGLU22*, *OsBGLU23*, and *OsBGLU31* in roots were the upregulated genes in both ‘Cr’ and ‘CrP’ treatments, while *OsBGLU6* and *OsBGLU26* were the upregulated genes in shoots under ‘CrP’ treatments (Figure 4). On the other hand, the *OsUGP1* expression value was also recorded higher under the ‘CrP’ application compared with ‘Cr’ treatments, which is associated with uridine diphosphate (UDP)-glucose pyrophosphorylases (UGPs), a crucial enzyme involved in cell wall biosynthesis [25]. Interestingly, two other cell-wall-remodeling β-amylase genes (*OsBMY1* and *OsBMY2*) were also expressed higher in ‘CrP’ treatments [26]. These findings reveal that ‘Cr’ treatments increased the synthesis of β-glucosidases (BGLU) and β-amylase enzymes, while Pro application improved uridine diphosphate (UDP)-glucose pyrophosphorylases enzymes coupled with β-glucosidases (BGLU) and β-amylase enzyme, which subsequently improved cell wall biosynthesis in rice plants under Cr stress.

Hemicellulose is another cell wall-strengthening component synthesized from sugar nucleotides by glycosyltransferases. These enzymes are type II transmembrane proteins with functional domains in the Golgi lumen from where the hemicellulose molecules are transported to the cell wall [27]. Under ‘Cr’ and ‘CrP’ treatments, *OsXTH29* and *OsXTH25* were the upregulated genes associated with the hemicellulose pathway of cell walls in the roots of rice plants (Figure 5). On the other hand, *OsXTH1*, *OsXTH9*, *OsXTH10*, *OsXTH16*, *OsGAUT3*, *OsGAUT19*, *OsGAUT28*, *OsGAUT12*, and *OsGAUT14* had higher *GEVF* values between ‘Cr’ and ‘CrP’ treatments. In both cases, several genes are associated with xyloglucan endotransglucosylase/hydrolase (XTH), which is a key enzyme in the process of plant cell wall remodeling, i.e., regulates the elasticity and ductility of cells and provides cell wall ductility without reducing the mechanical properties of the cell wall [28]. The XTH gene family is involved in various physiological processes in plants, especially in abiotic stress responses and cell elongation [29]. In the current findings, several XTH genes, i.e., *OsXTH1*, *OsXTH9*, *OsXTH10*, and *OsXTH16*, had higher *GEVFs* for ‘CrP’ treatments. In addition, several galacturonosyltransferase (GAUT)-associated genes, i.e., *OsGAUT3*, *OsGAUT19*, *OsGAUT28*, *OsGAUT12*, and *OsGAUT14*, had significantly higher *GEVF* values in rice seedlings exposed to Cr, which are involved in the biosynthesis of pectin and xylan (hemicellulose) in cell walls [30]. These results suggest that Cr stress alters the expression of genes associated with xyloglucan endotransglucosylase/hydrolase and galacturonosyltransferase enzymes associated with the hemicellulose pathway of cell walls; however, the application of Pro further improves the expression of these genes under Cr stress in rice plants.

Lignin is the second most abundant polymer found in nature after cellulose and plays a crucial role in the cell wall. The lignin biosynthesis pathway is a multistep process that begins in the cytosol with the synthesis of glycosylated monolignols from the amino acid phenylalanine. The monolignols are transported to the cell wall and their coupling generates the lignin polymer [31]. In the lignin pathway, *OsPRX37*, *OsPRX70*, and *OsPRX77* in roots and *OsPRX77* in shoots were the upregulated genes in both ‘Cr’ and ‘CrP’ treatments (Figure 6), while *OsPRX130* and *OsPRX137* were the only upregulated genes observed under ‘Cr’ treatments. Several genes in roots (*OsPME25* and *OsPGL27*) and shoots (*OsPME26*, *OsPGL9*, and *OsPLL12*) were significantly upregulated under all the treatments of ‘CrP’ compared with the ‘Cr’ exposure alone. In the lignin biosynthesis pathway, phenylalanine ammonialyase (PAL) is a critical enzyme involved in plant growth and the stress response [22], whereas PAL-associated genes, i.e., *OsPAL3* and *OsPAL5,* had higher *GEVF* values in rice plants for exogenous Pro compared with Cr treatments alone. During abiotic stresses, an increase in peroxidase (POX/PRX) activity causes the lignification and rigidification of cell walls, strengthening the cell walls [32]. For instance, *OsPOX1* has been reported as a cold-responsive gene in rice [33]. The results in the current study showed that *OsPRX130* and *OsPRX137* were the upregulated genes under ‘Cr’ treatments, while *OsPRX37*, *OsPRX70*, and *OsPRX77* were the upregulated genes in both ‘Cr’ and ‘CrP’ treatments associated with peroxidase. Similarly, *OsPOX1* and *OsPRX77* had higher *GEVF* values within ‘Cr’ and ‘CrP’ treatments. This suggests that Pro can improve the biosynthesis of phenylalanine ammonialyase, peroxidase, and pectin methylesterase enzymes and subsequently the synthesis of cell walls under Cr stress (22; 32).

Pectins are the major component of the primary cell wall, accounting for approximately 35% of primary cell walls in dicots and non-Graminaceous monocots. They are synthesized in the Golgi apparatus and subsequently transported to the cell wall, where they regulate cell wall properties such as extensibility and thickness [34]. Pectin polysaccharide is the major binding and accumulation site for HMs in the cell wall due to the presence of homogalacturonan in addition to rhamnogalacturonan-I and rhamnogalacturonan-II [35]. Pectin demethylation is carried out by pectin methylesterase (PME), which is an important process in cell walls to adsorb HMs and enhance their capacity for binding HMs [36]. The current findings showed that *OsPME20* was upregulated under ‘Cr’ treatments (Figure 7), while *OsPME25* and *OsPME26* had higher *GEVF* values in rice plants exposed to Cr in the presence of exogenous Pro. Pectate lyase (PGL)-responsible genes, i.e., *OsPGL27* and *OsPGL9*, were also expressed higher by the Pro application during Cr exposure, which depolymerizes pectins by catalyzing the eliminative cleavage of α-1,4-linked galacturonic acid. This indicates that exogenous Pro improves cell wall-synthesizing enzymes associated with pectin under Cr stress. In addition, it also proposes that the upregulation of cell wall biosynthesis genes reduces cell wall damage and maintains new cell wall synthesis as well. In one case, the exogenous application of Pro not only reduced the toxicity of Cr in rice plants but also enhanced their accumulation in rice tissues [14]. Proline-rich proteins are cell wall- and cell membrane-anchored factors involved in cell wall maintenance and stress-induced fortification [37]. So, the application of Pro under Cr stress would reduce cell wall damage and improve cell wall synthesis.

## 5. Conclusions

The present study used rice microarray data to identify the core genes involved in cell wall modification in rice plants under Cr stress with or without exogenous Pro. Several key genes were identified on the basis of their upregulation under ‘Cr’ and/or ‘CrP’ treatments and on the *GEVF* values between ‘Cr’ and/or ‘CrP’ treatments. The identified genes provided in this article can open new doors toward the Cr-stress response in rice plants, specifically related to the cell wall. Therefore, further experimental work should be carried out on these genes for improving stress tolerance in rice during Cr exposure.

## Data Availability

The data is available at Supplementary Materials and the public databases mentioned in the study.

## Supplementary Materials

The following supporting information can be downloaded at: https://www.mdpi.com/article/10.3390/toxics12010004/s1, Table S1: Sequence of forward and reverse primers used in gene expression analysis; Table S2: Total number of DEGs detected in rice seedlings under the three effective concentrations of Cr with and without Pro treatment; Supplementary Data S1: Enzyme name, gene name, chromosome locus, and Cr-concentration-dependent up/downregulation of genes in roots and shoots.
