# Supplementary material for: Identification of the Key Genes Involved in Proline-Mediated Modification of Cell Wall Components in Rice Seedlings under Trivalent Chromium Exposure
Source: Toxics. 2023 Dec 19;12(1):4. doi: 10.3390/toxics12010004 (PMC10818556; doi:10.3390/toxics12010004)
Supplement: Supplementary file 1 [file toxics-12-00004-s001.zip › Supporting information S1.pdf]

Table S1: Sequence of forward and reverse primers used in gene expression analysis

Enzyme	Gene symbol	MSU ID	Primer sequences(5'-3')	Amplicon size (bp)
β-glucosidase	OsBGLU3	LOC_Os01g59840.1	F-GCTTGGTTCTTCGTGGACAT<br>R-GTTGCCGCTTGCTCTTGA	151
	OsBGLU6	LOC_Os03g11420.1	F-GTGCTGCTCCACCTCTACTG<br>R-CTGGTGGGCTGTTGTACCTT	574
	OsBGLU18	LOC_Os04g43410.1	F-TTACACCGACGCAGAGGAT<br>R-CGAAGTAGCCACGAACATCA	116
	OsBGLU26	LOC_Os07g46280.1	F-GGAACCTGACACCGACCAGTT<br>R-GCACCATCGTCTATCGCCTTCT	298
	OsGH5BG	LOC_Os10g22520.1	F-TCCTCCTCTTCCTCCTCCTCT<br>R-CTGCGTCACCGACTTGAATTG	221
β-amylase	OsBMY1	LOC_Os07g35880.1	F-CAGGACTGCCATCCAGTTGT<br>R-CGAGGCCAACCTCTATGTCC	104
	OsBMY6	LOC_Os10g41550.1	F-GGTGTTGGAGGAGATGAAGTC<br>R-GAGGTAGCCGCAGAATGTG	178
Xyloglucan endotransglucosylase/hydrolase	OsXTH6	LOC_Os04g51450.1	F-GGTCGGCGTTCAAGTCCAAGAC<br>R-TCGTCGTGGGTGTTCCAATCCT	136
	OsXTH9	LOC_Os04g51460.1	F-GTTCCAGTCCAAGAGCGAGTA<br>R-CTGCGACGACAAGTAGAAGGT	106
	OsXTH11	LOC_Os06g48160.1	F-GTCGTTGCCTTCTACCTGTC<br>R-AGAGGACGGAGTAGCGATG	205
	OsXTH23	LOC_Os02g46910.1	F-CCGCTACTTCACCGAGACCTT<br>R-CGGCGAACACCAGCAAGAAC	227
Galacturonosyltransferase	OsGATL6	LOC_Os02g50600.1	F-TGGCAGCGGTTCATTCATC<br>R-TTAGCAAGCACAGCAGACTC	229
Fucosyltransferase	OsFUT11	LOC_Os08g36840.1	F-GACCGCAACCTCATCATCC<br>R-CCTTGTTCATCCACTTCTCCTT	274
Phenylalanine ammonia-lyase	OsPAL2	LOC_Os02g41650.1	F-ATGAAGCCGAAGCAGGACAG<br>R-GGAGATGAGACCAAGCGAGTT	462
	OsPAL6	LOC_Os04g43800.1	F-CTCTGCTCCAGGGGTACTCT<br>R-GCTTCTTCGCCAGCATCATG	513

	OsPAL7	LOC_Os05g35290.1	F-CACATCTTGGAAGGCAG<br>R-GAGAGGTTGGAGGGCAG	365
4-coumarate: CoA ligase	Os4CL1	LOC_Os08g14760.1	F-AGGACGACGAGAAGGTGTT<br>R-ATGGTGTTGAGCGAGTAGATG	217
	Os4CL5	LOC_Os08g34790.1	F-CTCCACTCCATCCTCCTCTG<br>R-CGATGTCCTGAAGCTCCTTG	238
Peroxidase	OsPRX77	LOC_Os06g16350.1	F-GCTCTGATGCTTCGTCTTC<br>R-CATTCTGTTCTGCCTTCTTCT	112
	OsPRX109	LOC_Os07g47990.1	F-GCGTCACTGCTCAGGCTTCACT<br>R-CCTCGACCTGCGCCTTGATGTT	166
Pectinesterase	OsPME3	LOC_Os01g21034.1	F-ACTCGAACAAGAGGTACGTG<br>R-CCGAAGATGAAGTCGATGGT	388
	OsPME7	LOC_Os01g65790.1	F-CGCTCAAGACGCTCTACTACG<br>R-GGTATCCACTTGTCACCCTGT	154
	OsPME23	LOC_Os07g49100.1	F-ACGTGAACATGATAGGCGAC<br>R-CTGAAGAACAACCTGCGAGT	332
Polygalacturonases	OsPGL7	LOC_Os01g66710.1	F-GAACGTGACGGTGAAGACGA<br>R-GTGGAGTCCGAGAACGTGAC	114
	OsPGL9	LOC_Os05g46510.1	F-TCCGTGATGCGGCTGGTTGTTG<br>R-GCCTGCGTGTCGTTGGTAATGC	140
Pectate lyase	OsPLL1	LOC_Os01g36620.1	F-CGTCCACCACTGCTTCTTC<br>R-TGAACACCGCCTTCCTCTC	185
	OsPLL4	LOC_Os04g05050.1	F-GCAGGTCACGATAGCATTCA<br>R-ATGGTTGGCTCAGCACTTC	144
Glyceraldehyde-3-phosphate dehydrogenase	*OsGAPDH1*	LOC_Os08g03290.1	F-GACAGCAGGTCGAGCATCTTC<br>R-CAGGCGACAAGCTTGACAAAG	74

Table S2: Total number of DEGs detected in rice seedlings under the three effective concentrations of Cr with and without Pro treatment

Cr treatments	Total number of DEGs	Up-regulated DEGs (Roots/Shoots)	Down-regulated DEGS (Roots/Shoots)

12 mg Cr/L	143	17/14	50/3
24 mg Cr/L	106	20/14	69/3
40 mg Cr/L	121	22/23	63/13

Cr treatments with Proline application	Total number of DEGs	Up-regulated DEGs (Roots/Shoots)	Down-regulated DEGS (Roots/Shoots)
12 mg Cr/L	106	15/8	56/27
24 mg Cr/L	107	11/7	65/24
40 mg Cr/L	140	31/26	61/22
